# Draft Genome Sequence of *Mesosutterella multiformis* JCM 32464^T^, a Member of the Family *Sutterellaceae*, Isolated from Human Feces

**DOI:** 10.1128/MRA.00478-19

**Published:** 2019-06-13

**Authors:** Nao Ikeyama, Moriya Ohkuma, Mitsuo Sakamoto

**Affiliations:** a Microbe Division/Japan Collection of Microorganisms, RIKEN BioResource Research Center, Tsukuba, Ibaraki, Japan; b PRIME, Japan Agency for Medical Research and Development (AMED), Tsukuba, Ibaraki, Japan; University of Maryland School of Medicine

## Abstract

Here, we report the draft genome sequence of *Mesosutterella multiformis* JCM 32464^T^, a new member of the family *Sutterellaceae* that was isolated from human feces. The genome assembly comprised 2,621,983 bp, with a G+C content of 56.9%. This genomic analysis will be useful for understanding the metabolic activities of this asaccharolytic bacterium.

## ANNOUNCEMENT

Along with the development of culture methods, novel species have been isolated from human feces, and it is important to characterize and clarify their role in the human gut. Sutterella spp. are commonly present in the healthy human gut, but it has been reported that there is an association between *Sutterella* spp. and gastrointestinal (GI) disturbances in children with autism (1) and mild proinflammatory capacity in the human GI tract (2). Most *Sutterella* spp. are asaccharolytic, and metabolic requirements for their growth are unclear; this makes cultivation difficult. Recently, we isolated Mesosutterella multiformis JCM 32464^T^ from human feces (3). This strain is a new member of the family *Sutterellaceae*; it is phylogenetically located between the genera Parasutterella and Sutterella. In the 16S rRNA gene sequence analysis, M. multiformis JCM 32464^T^ showed relatively low similarity to Sutterella stercoricanis CCUG 47620^T^ (92.6%), Sutterella wadsworthensis WAL 7877 (92.4%), Sutterella parvirubra YIT 11816^T^ (92.1%), and Parasutterella secunda YIT 12071^T^ (91.8%). We analyzed the draft genome sequence of *M. multiformis* JCM 32464^T^ to understand the role of this strain in the human gut.

*M. multiformis* JCM 32464^T^ was grown on brucella blood agar with hemin and menadione for 4 days at 37°C under a H_2_/CO_2_/N_2_ (1:1:8, vol/vol/vol) gas mixture (3). The genomic DNA of *M. multiformis* JCM 32464^T^ was extracted using a Genomic-tip 100/G kit (Qiagen) and by lysing bacterial cells with labiase (5.0 mg · ml^−1^; Cosmo Bio). The whole genome of *M. multiformis* JCM 32464^T^ was sequenced using the PacBio RS II sequencing system (Pacific Biosciences) at TaKaRa Bio, Inc. (Shiga, Japan). For PacBio sequencing, the library was prepared using a SMRTbell template prep kit 1.0 (Pacific Biosciences), followed by single-molecule real-time (SMRT) sequencing. After we filtered the reads using PreAssembler Filter version 1 (minimum subread length, 500 bp; minimum polymerase read quality, 0.80; minimum polymerase length, 100 bp) in SMRT Analysis version 2.3.0 (4), a total of 81,549 reads (340-fold coverage) with an average length of 13,355 bp were assembled *de novo* using Hierarchical Genome Assembly Process version 3.0 (HGAP3.0) in SMRT Analysis version 2.3.0 (4), resulting in 2 contigs with an *N*_50_ length of 1,920,098 bp. This assembly resulted in a draft genome sequence of 2,621,983 bp with a G+C content of 56.9%, containing 2,311 protein-coding sequences (CDSs), 55 tRNAs, and 18 rRNAs detected using Rapid Annotations using Subsystem Technology (RAST) version 2.0 (5), the DDBJ Fast Annotations and Submission Tool (DFAST) version 1.1.0 (6), and the Kyoto Encyclopedia of Genes and Genomes (KEGG) release 90.0 (7). Default parameters were used for all software except where otherwise noted.

The genome of *M. multiformis* JCM 32464^T^ lacked complete carbohydrate metabolic pathways, as shown by the biochemical characteristics. However, *M. multiformis* JCM 32464^T^ was predicted to possess a heme biosynthesis pathway, which is coupled to electron transport chains for energy generation (8, 9). The energy generated by this pathway might be usable for the growth of *M. multiformis*. It has been reported that protoporphyrinogen IX oxidase (PPO; encoded by *hemG*) (HemG; EC 1.3.5.3) plays an important role in this pathway (9). The genome of *M. multiformis* JCM 32464^T^ contained an *hemG* gene (MESMUL_07990). Furthermore, fumarate reductase (EC 1.3.5.4), nitrate reductase (EC 1.7.5.1), and F-type ATPase were predicted. We presume that these enzymes are utilized along with HemG and menaquinones for electron transfer reactions and ATP generation; a trace amount of ATP will be yielded by the proton motive force under anaerobic conditions. *M. multiformis* JCM 32464^T^ possessed menaquinones (3).

Our data indicate one of the energy acquisition mechanisms of asaccharolytic bacteria, and the genome information for this species will lead to further study of the human gut microbiome and host-microbe symbiosis.

### Data availability.

The draft genome sequence of *M. multiformis* JCM 32464^T^ has been deposited in DDBJ/EMBL/GenBank under the accession numbers BGZJ01000001 and BGZJ01000002. Raw sequence reads have been deposited in the NCBI Sequence Read Archive under BioProject number PRJDB7181 and run number DRR177539.
